# CDDO-Me: A Novel Synthetic Triterpenoid for the Treatment of Pancreatic Cancer

**DOI:** 10.3390/cancers2041779

**Published:** 2010-10-13

**Authors:** Dorrah Deeb, Xiaohua Gao, Ali S. Arbab, Kenneth Barton, Scott A. Dulchavsky, Subhash C. Gautam

**Affiliations:** 1 Department of General Surgery, Henry Ford Health System, Detroit, MI 48202, USA; E-Mails: ddeeb1@hfhs.org (D.D); tgao1@hshs.org (X.G.); sdulcha1@hfhs.org (S.A.D); 2 Department of Diagnostic Radiology, Henry Ford Health System, Detroit, MI 48202, USA; E-Mail: sali1@hfhs.org (A.S.A.); 3 Department of Radiation Oncology, Henry Ford Health System, Detroit, MI 48202, USA; E-Mail: kbarton1@hfhs.org (K.B.)

**Keywords:** pancreatic cancer, CDDO-Me, apoptosis, Akt/mTOR signaling pathway

## Abstract

Pancreatic ductal adenocarcinoma (PDA) is one of the most lethal human malignancy with dismal prognosis and few effective therapeutic options. Novel agents that are safe and effective are urgently needed. Oleanolic acid-derived synthetic triterpenoids are potent antitumorigenic agents, but their efficacy or the mechanism of action for pancreatic cancer has not been adequately investigated. In this study, we evaluated the antitumor activity and the mechanism of action of methyl-2-cyano-3,12-dioxooleana-1,9(11)-dien-28-oate (CDDO-Me), a oleanane-derived synthetic triterpenoid for human pancreatic cancer cell lines. CDDO-Me inhibited the growth of both K-ras mutated (MiaPaca2, Panc1 and Capan2) and wild-type K-ras (BxPC3) pancreatic cancer cells at very low concentrations. The growth inhibitory activity of CDDO-Me was attributed to the induction of apoptosis characterized by increased annexin-V-FITC binding and cleavage of PARP-1 and procaspases-3, -8 and-9. In addition, CDDO-Me induced the loss of mitochondrial membrane potential and release of cytochrome C. The antitumor activity of CDDO-Me was associated with the inhibition of prosurvival p-Akt, NF-κB and mammalian target of rapamycin (mTOR) signaling proteins and the downstream targets of Akt and mTOR, such as p-Foxo3a (Akt) and p-S6K1, p-eIF-4E and p-4E-BP1 (mTOR). Silencing of Akt or mTOR with gene specific-siRNA sensitized the pancreatic cancer cells to CDDO-Me, demonstrating Akt and mTOR as molecular targets of CDDO-Me for its growth inhibitory and apoptosis-inducing activity.

## 1. Introduction

Pancreatic ductal adenocarcinoma (PDA) accounts for more than 90% of all exocrine pancreatic cancers and is the fourth most common cause of cancer-related deaths in the United States [1]. It is more common in elderly people, and the risk increases with age. Despite considerable progress in understanding the biology of PDA and advances in therapeutic interventions, this malignancy has remained almost uniformly lethal with an estimated annual incidence of 43,140 new cases approximating 36,800 annual deaths [2].

The lack of symptoms and screening techniques for early detection, aggressive metastatic behavior, and resistance to conventional chemotherapy and radiotherapy regimens render pancreatic cancer with the worst prognosis of any major malignancy and an overall five-year survival rate of <5% [3,4]. Surgical resection is the only curative modality; however, 50% of patients have locally advanced and metastatic disease at the time of diagnosis, precluding curative surgical intervention. Most chemotherapeutic agents, including gemcitabine, have little effect on neoplastic progression and survival [5,6]. Thus, there is tremendous need for more active agents and novel strategies for the treatment of pancreatic cancer.

Triterpenes or triterpenoids are members of a larger family of structurally related compounds known as cyclosqualenoids that are widely distributed in the plant kingdom [7]. Oleanolic acid and ursolic acid are naturally occurring triterpenoids that have been used in traditional medicine for centuries as antibacterial, antifungal, anti-inflammatory and anti-cancer agents [8,9]. Synthetic derivative of oleanolic acid: 2-cyano-3,12-dioxooleana-1,9(11)-dien-28-oic acid (CDDO) and its C-28 methyl ester (CDDO-Me) and C-28 imidazole (CDDO-Im) derivatives have shown strong anti-inflammatory [10,11] and anti-proliferative activity against diverse types of tumor cell lines *in vitro* [12,13,14] and tumor development *in vivo* [15,16]. Although the mechanisms of the anticancer effects of CDDOs are not fully understood, cancer cell differentiation, apoptosis and modulation of MAPK (Erk1/2), NF-κB, TGF-β/Smad and PPARγ signaling pathways contribute to the antitumor activity of CDDOs [17,18,19,20].

We have previously shown that CDDO-Me inhibits the growth of hormone-sensitive and -refractory human prostate cancer cell lines *in vitro* and *in vivo* by inducing apoptosis [21,22]. CDDO-Me inhibited the prosurvival Akt, NF-κB and mammalian target of rapamycin (mTOR) signaling proteins in prostate cancer cells. The antitumor activity of CDDOs for pancreatic cancer has not been tested adequately. In the present study, the response of human pancreatic cancer cell lines to CDDO-Me was investigated. The results demonstrate that CDDO-Me inhibited the growth of pancreatic cancer cells by inducing apoptosis through the activation of procaspases -3, -8, -9 and mitochondrial depolarization. Furthermore, induction of apoptosis was associated with the inhibition of the Akt/mTOR signaling axis and downstream mediators of this signaling pathway.

## 2. Results

### 2.1. CDDO-Me Inhibits the Growth of Pancreatic Carcinoma Cells

**Figure 1:**
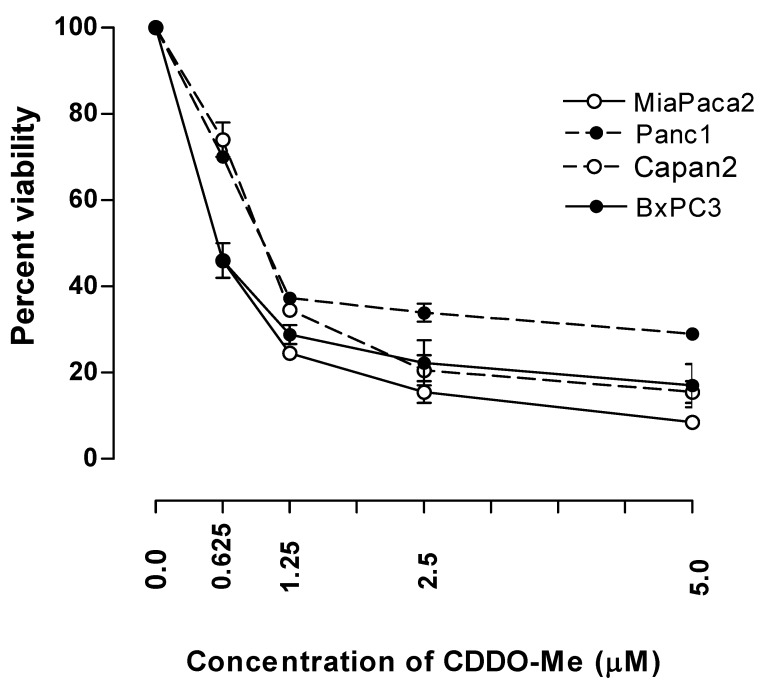
CDDO-Me inhibits the growth of pancreatic cancer cells. 1 × 10^4^ MiaPaca2, Panc1, Capan2 or BxPC3 cells were treated with CDDO-Me at concentrations ranging from 0 to 5 µM for 72 h in triplicate in a 96-well microtiter plate. Cell viability was measured by MTS assay using the CellTiter AQueous assay system from Promega. Data are presented as percent reduction in viability obtained in three independent experiments.

The effect of CDDO-Me on the growth of human pancreatic cancer cells (MiaPaca2, Capan2, Panc1 and BxPC3) was examined using CellTiter 96® AQueous Non-Radioactive Cell Proliferation Assay (MTS). As shown in Figure 1, a measurable reduction in viability of all cell lines was observed at 0.625 µM CDDO-Me (21% to 52% inhibition); however, significant reduction in viability in the majority of the cell lines occurred at 1.25 µM (by 70% to 76%). In the case MiaPaca2, Capan2 and BxPC3 cells, there was nearly 90% reduction in viability at 5 µM CDDO-Me. Panc1 cells were less sensitive to CDDO-Me compared to the other cell lines, showing 50% to 73% growth inhibition at concentrations of 1.25 to 5 µM. On the other hand, non-transformed prostate epithelial BPH1 cells, normal fibroblasts and splenic lymphocytes were significantly more resistant to CDDO-Me at concentrations of 2.5 to 5 µM compared to pancreatic cancer cells. For instance, there was 27% reduction in the viability of BPH1 cells at 2.5 µM CDDO-Me compared to 75% to 80% reduction in most of the pancreatic cancer cell lines. Fibroblasts were resistant to CDDO-Me up to 10 µM, while splenic lymphocytes showed modest reduction in viability (24%) at 10 µM CDDO-Me.

### 2.2. CDDO-Me Induces Apoptosis in Pancreatic Cancer Cells

**Figure 2:**
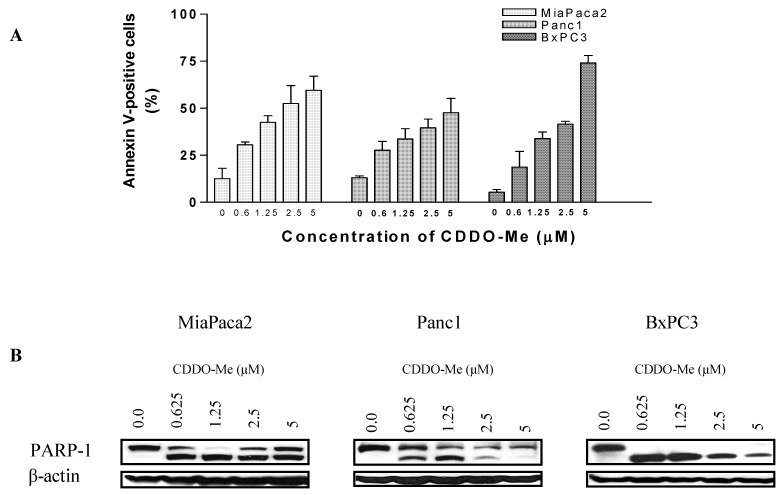
Treatment with CDDO-Me increases annexin V-FITC binding and cleavage of PARP-1. (**A**) MiaPaca2, Panc1 and BxPC3 cells were treated with CDDO-Me at concentrations of 0.625 to 5 µM for 24 h. Cells were then incubated with 5 μL of annexin V-FITC reagent plus 5 µL of PI for 30 min at room temperature and analyzed by flow cytometry. (**B**) Cleavage of PARP-1 in pancreatic cancer cells by CDDO-Me was detected by immunoblotting. Similar results were obtained in two independent experiments.

**Figure S1:**
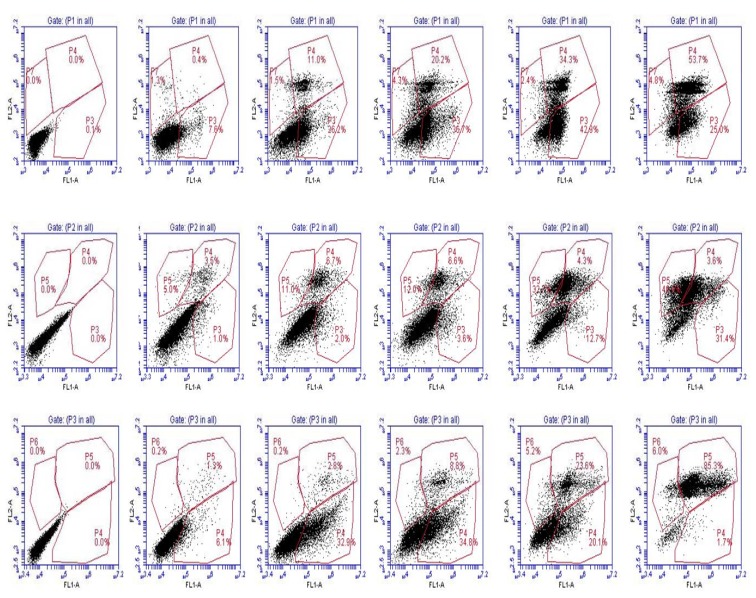
FACS analysis of pancreatic cancer cells stained with annexin V-FITC and PI.

Whether inhibition of growth of pancreatic cancer cells by CDDO-Me was due to induction of apoptosis was investigated by measuring the binding of annexin V-FITC and cleavage of PARP-1. As shown in Figure 2A and Supplementary Figure S1, a small percentage of untreated MiaPaca2, Panc1 and BxPC3 (12%, 13% and 5%, respectively) bound annexin V-FITC. In contrast, the percentage of annexin V-FITC binding cells markedly increased after treatment with CDDO-Me (0.625–5 µM) for 24 h (MiaPaca2, 32–59%; Panc1, 28–47% and BxPC3, 19–74%).

The induction of apoptosis was confirmed by the cleavage of PARP-1 as identified by the disappearance of the 116 kDa native protein and the emergence of an 89 kDa cleaved PARP-1 fragment in MiaPaca2, Panc1 and BxPC3 cells treated with CDDO-Me (Figure 2B). Thus, increase in annexin V-FITC-binding and the cleavage of PARP-1 suggest that the growth inhibitory activity of CDDO-Me against pancreatic cancer cells is due to induction of apoptosis.

### 2.3. CDDO-Me Induces Cleavage of Procaspases, Mitochondrial Depolarization and Release of Cytochrome C

**Figure 3:**
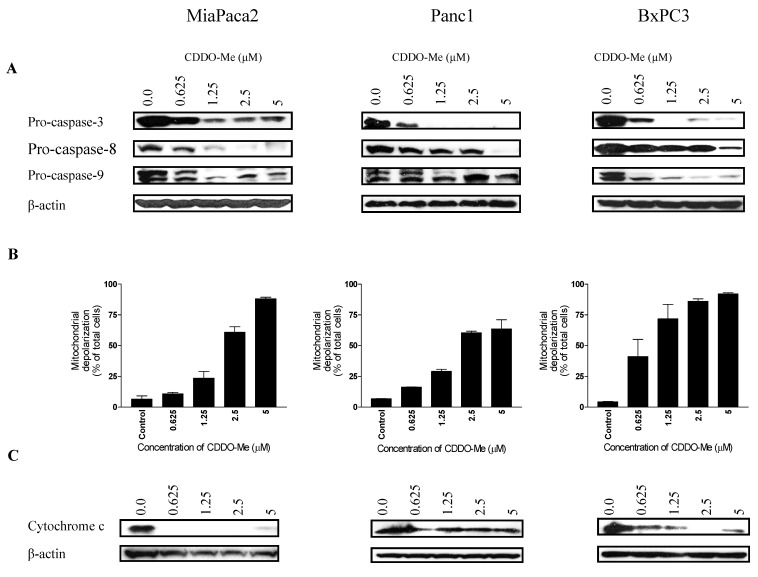
Effect of CDDO-Me on procaspases and mitochondrial integrity. MiaPaca2, Panc1 and BxPC3 cells were treated with CDDO-Me at 0.625 to 5 µM for 24 h and analyzed for the cleavage of procaspase-8, -3, -9 (**A**), mitochondrial depolarization (**B**) or release of cytochrome C from mitochondria (**C**). Similar results were obtained in two separate experiments.

To investigate whether induction of apoptosis by CDDO-Me was associated with the activation of procaspases, we measured the processing of procaspases associated with the extrinsic (procaspases-8 and -3) and intrinsic (procaspase-9) pathways of apoptosis. Western blot analysis of cell lysates of MiaPaca2, Panc1 and BxPC3 cells treated with CDDO-Me showed processing of these procaspases in a dose-related manner (Figure 3A). Procaspase-3 was significantly to completely processed in each of the three cell lines at concentrations of 0.625 µM and above. Procaspase-9 was most affected at 1.25 μM and higher concentrations of CDDO-Me. The effect on procaspase-8 was variable with complete reduction in MiaPaca2 cells at 1.25–2.5 μM while BxPC3 cells were most affected at 5 μM CDDO-Me. In Panc1 cells, there was significant reduction in procaspase-8 at 2.5 µM and above. These data indicated that CDDO-Me activates procaspases associated with both pathway of apoptosis.

**Figure S2:**
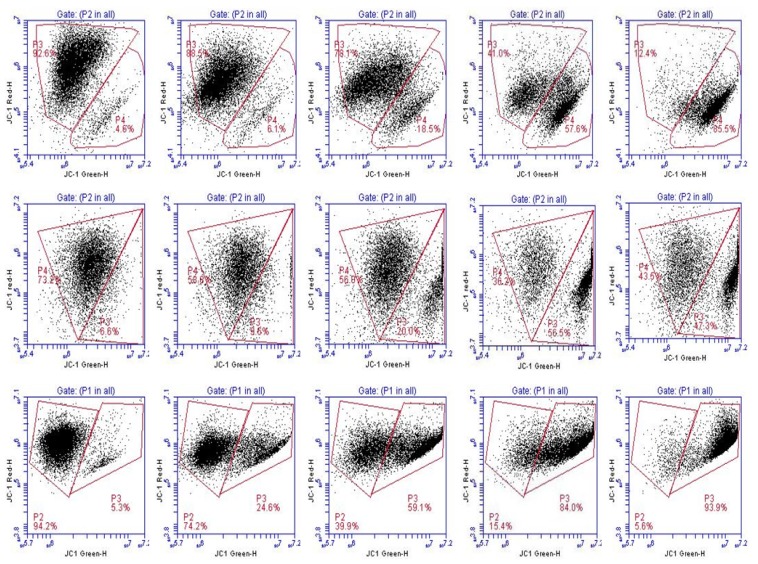
FACS analysis of pancreatic cancer cells loaded with mitochondrial membrane potential probe JC-1.

To further investigate the involvement of the mitochondrial ‘intrinsic’ pathway of apoptosis, we evaluated mitochondrial depolarization and release of cytochrome C from mitochondria in cells treated with CDDO-Me. Mitochondrial depolarization was determined from the fluorescent shift of cells loaded with mitochondrial-potential JC-1 dye. As shown in Figure 3B, there was significant change in mitochondrial potential after treatment with CDDO-Me for 24 h. The percentage of MiaPaca2 cells with green fluorescence increased from 6% (0 CDDO-Me) to 9%, 23%, 60% and 87% at 0.625, 1.25, 2.5, 5 µM CDDO-Me, respectively. The percentage of Panc1 cells with green fluorescence was 7%, 16%, 28%, 59% and 63% of cells at 0, 0.625, 1.25, 2.5, and 5 µM CDDO-Me, respectively. The BxPC3 cell line also showed an increase in cells with green fluorescence at increasing concentration of CDOO-Me (e.g., 4%, 41%, 71%, 86% and 92% at 0, 0.625, 1.25, 2.5, and 5 µM CDDO-Me, respectively). A representative experiment showing FACS analysis of cells loaded with mitochondrial-potential probe JC-1 is shown in Supplementary Figure S2.

Treatment with CDDO-Me also induced the release of cytochrome C from mitochondria in each of the cell lines (Figure 3C). MiaPaca2 cells demonstrated complete loss of cytochrome C from the mitochondria even at the lowest concentration of 0.625 µM followed by BxPC3 cells showing more than 80% loss at 0.0625 µM and above. Panc1 cells showed about 50% reduction at all concentrations of CDDO-Me. Thus, the cleavage of procaspase-9, mitochondrial depolarization and release of cytochrome C from mitochondria demonstrated the involvement of mitochondrial ‘intrinsic’ pathway of apoptosis in apoptotic death of pancreatic cancer cells by CDDO-Me.

### 2.4. CDDO-Me Inhibits Akt, NF-κB and mTOR and Downstream Signaling Proteins

**Figure 4:**
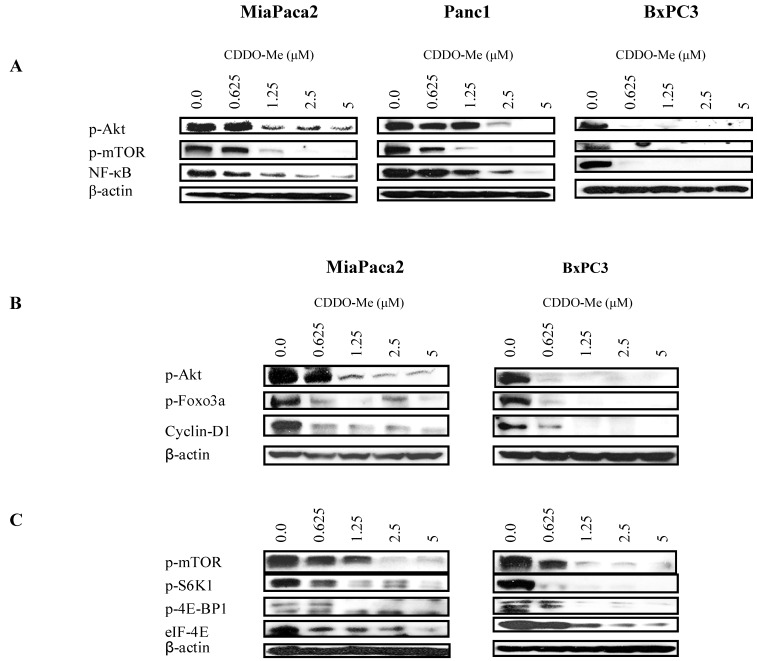
CDDO-Me inhibits p-Akt, NF-κB (p65) and p-mTOR expression in pancreatic cancer cells. (**A**). MiaPaca2, Panc1 or BxPC3 cells were treated with CDDO-Me at concentrations of 0.625 to 5 µM for 24 h and analyzed for p-Akt, p-mTOR and NF-κB levels by immunoblotting. (**B**, **C**). Effect of CDDO-Me on downstream targets of p-Akt (p-Foxo3a and cyclin D1) and p-mTOR (p-S6K1, p-eIF-4E, p-4E-BP1).

Akt, mTOR and NF-κB are major antiapoptotic signaling proteins that confer survival advantage and resistance of cancer cells to various forms of anticancer therapies. We determined whether induction of apoptosis in pancreatic cancer cells by CDDO-Me involved inhibition of Akt, mTOR and NF-κB. For this purpose, cancer cells were treated with CDDO-Me (0.625 to 5 μM) for 24 h and cell lysates analyzed for p-Akt, p-mTOR and NF-κB (p65) by Western blotting (Figure 4A). Treatment with CDDO-Me significantly to completely abolished p-Akt in MiaPaca2 cells at 0.625 μM and above. In Panc1 cells, p-Akt was 30 to 40% reduced at concentrations of 0.625 and 1.25 μM but was completely inhibited at 2.5–5 μM CDDO-Me. The effect of CDDO-Me on p-mTOR and NF-κB was almost identical to that on p-Akt, *i.e.,* more than 80% reduction in the levels of these proteins at 1.25–5 μM CDDO-Me. The levels of basal Akt, mTOR or NF-κB were not affected by CDDO-Me.

To further explore modulation of the Akt/mTOR signaling axis, the effect of CDDO-Me on expression of downstream mediators of Akt and mTOR signaling pathways was evaluated in MiaPaca2 and BxPC3 cells (Figure 4B). CDDO-Me inhibited p-Foxo3a and cyclin D1, the downstream intermediary targets of activated Akt, in both cell lines at the low concentration of 0.625 μM. The levels of mTOR pathway mediators: p-S6K1, p-eIF-4E, and p-4E-BP-1, were also either reduced or completely inhibited by CDDO-Me in a concentration-related manner (Figure 4C). Together, these data suggest that the inhibition of antiapoptotic Akt, mTOR and NF-κB might be necessary for induction of apoptosis by CDDO-Me in pancreatic cancer cells.

### 2.5. Akt and mTOR Are Relevant Targets of CDDO-Me

**Figure 5:**
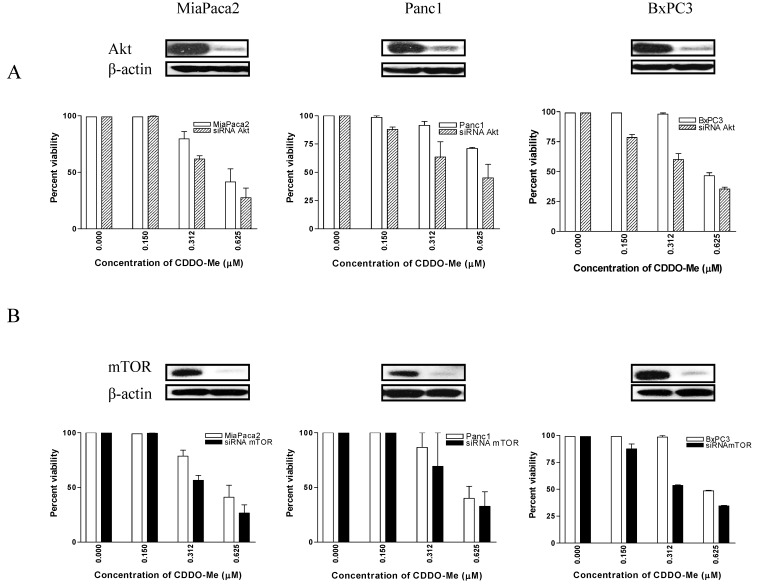
Akt and mTOR are molecular targets of CDDO-Me. MiaPaca2, Panc1 and BxPC3 were transfected with siRNA-Akt (A) or siRNA-mTOR (B). The ablation of Akt and mTOR in transfected cells was confirmed by immunoblotting (insets) and susceptibility to low concentrations of CDDO-Me was measured by MTS assay.

Since CDDO-Me inhibited both p-Akt and p-mTOR in pancreatic cancer cells we determined whether the Akt/mTOR signaling axis regulates response of these cancer cells to CDDO-Me. This was investigated by knocking down the levels of Akt and mTOR in MiaPaca2, Panc1 and BxPC3 cells with gene specific siRNA. Thus, cells were transfected with either siRNA-Akt or siRNA-mTOR and their response to low concentrations of CDDO-Me was measured. As shown in Figure 5, transfection with gene-specific siRNA completely abolished the expression of Akt and mTOR in each cell line (insets). As expected, control cells (non-transfected) were not very responsive to CDDO-Me at 0.156–0.312 µM (Figure 5A, B). In contrast, BxPC3 and Panc1 cells in which Akt has been silenced showed increased susceptibility to CDDO-Me at 0.156 µM and 0.312 µM compared to control cells. Transfected MiaPaca2 cells showed increased susceptibility only at 0.312 μM CDDO-Me. There was some improvement in the response of transfected cells treated with 0.625 µM CDDO-Me compared to control cells. The knock-down of mTOR improved the susceptibility of each of the cell lines at 0.312 µM but not at 0.156 μM CDDO-Me (Figure 5B). Again, there was some improvement in the response of cells treated with 0.625 µM CDDO-Me compared to control cells. Transfection with a non-targeting siRNA (both Akt and mTOR) did not sensitize cancer cells to CDDO-Me (not shown). Overall, these data indicated that Akt and mTOR are the molecular targets of CDDO-Me in pancreatic cancer cells.

### 2.6. Discussion

Lack of effective therapeutic options for pancreatic cancer underscores the need for developing novel agents to treat this highly lethal disease. Promotion of apoptosis in pancreatic cancer cells could potentially lead to the regression and improved prognosis of this chemotherapy refractory malignancy. Synthetic triterpenoids derived from oleanolic acid are potent antiproliferative and antitumorigenic agents that induce apoptosis in a wide range of human cancer cells including glioblastoma, osteosarcoma, leukemia, multiple myeloma, breast cancer, lung cancer, and prostate cancer cells [12,13,14,17,18,19]. The anticancer activity of synthetic triterpenoids for pancreatic cancer cells has not been adequately investigated. The present study demonstrated that CDDO-Me strongly inhibits the growth of pancreatic cancer cells by inducing cytotoxicity. Killing of pancreatic cancer cells by CDDO-Me was associated with an increase in annexin-V-FITC binding, cleavage of PARP-1 and procaspases, mitochondrial depolarization, release of cytochrome C, and inhibition of prosurvival/progrowth signaling proteins such as p-Akt, NF-κB and p-mTOR. The response of pancreatic cancer cells to CDDO-Me was independent of K-ras mutation, since both K-ras mutant (MiaPaca2, Panc1 and Capan2) and wild-type K-ras containing BxPC3 cells were equally sensitive to CDDO-Me. This however needs to be validated by testing additional wild-type K-ras positive pancreatic cancer cells.

Synthetic CDDOs have been shown to induce caspase-dependent and –independent apoptosis in cancer cells including pancreatic cancer cells [17,23]. In all of the PDA cell lines we tested, the growth inhibitory effect of CDDO-Me was attributed to the induction of apoptosis as determined by the binding of annexin V-FITC and cleavage of PARP-1 and procaspases-3, -8, and -9. The induction of mitochondrial depolarization and release of cytochrome C by CDDO-Me further supported the notion that induction of apoptosis is part of the mechanism by which CDDO-Me inhibits the growth of pancreatic cancer cells. Two major pathways of apoptotic cell death have been identified, namely the receptor-mediated (extrinsic) and mitochondrial (intrinsic) pathway of apoptosis. In both cases, caspases, a family of cysteine proteases, play an important role in apoptotic cell death [24]. In the extrinsic pathway, the binding of death ligands (e.g., TNF-α, FasL or TRAIL *etc*.) to their cognate receptors activates initiator caspase-8 which then cleaves and activates effector caspases -3, -6, and -7 leading to apoptosis [25]. In the intrinsic pathway induced by chemotherapeutic agents, cytochrome C released from mitochondria in conjunction with Apaf-1 causes the activation of initiator caspase-9. Activated caspase-9 then activates effector caspases -3, -6, and -7 [24]. CDDO-Me caused the cleavage of the most apical initiator procaspase-8 and the effector procaspase-3 in pancreatic cancer cells, indicating that extrinsic pathway of apoptosis is involved in the apoptotic death of pancreatic cancer cells by CDDO-Me. Whether CDDO-Me increases the expression of death receptors DR4 and DR5 remains to be determined. CDDO-Me also induced mitochondrial depolarization, release of cytochrome C from mitochondria and the cleavage of procaspase-9, indicating that the intrinsic pathway of apoptosis is also activated by CDDO-Me. The activation of both pathways of apoptosis suggests that CDDO-Me is potentially a potent chemotoxic agent for the treatment of prostate cancer.

PI3K/Akt/mTOR and Akt/NF-κB are major antiapoptotic/prosurvival signaling pathways that are frequently hyperactivated in many cancers [26,27]. Activated p-Akt promotes cell growth and survival by inactivating downstream substrates such as Bad, procaspase-9, and Forkhead transcription factors [28,29]. mTOR, a serine-threonine kinase controls cell growth, survival, division and motility [30]. CDDO-Me inhibited p-Akt, NF-κB and p-mTOR in pancreatic cancer cells. CDDO-Me also reduced/inhibited the downstream targets of Akt (Foxo3a and cyclin D1) and mTOR (p-S6K1, p-eIF-4E, p-4E-BP1). The inhibition of Akt/mTOR signaling pathway and its downstream intermediates suggested that the inhibition of these antiapoptotic proteins is critical for the induction of apoptosis by CDDO-Me in pancreatic cancer cells.

Since CDDO-Me inhibited p-Akt and p-mTOR, we investigated the functional relevance of these signaling proteins in mediating the antitumor activity of CDDO-Me. Selective inhibition of Akt or mTOR with gene-specific siRNA increased the sensitivity of pancreatic cancer cells to CDDO-Me at concentrations which were otherwise inactive. Whether overexpression of Akt or mTOR will render tumor cells resistant to CDDO-Me remains to be tested. Thus, these experiments showed that Akt and mTOR are major targets of CDDO-Me for suppressing growth and inducing apoptosis in pancreatic cancer cells. The next obvious step is to determine the efficacy of CDDO-Me *in vivo* in an orthotopic xenograft model and whether Akt and mTOR are relevant molecular targets of CDDO-Me *in vivo* as well.

## 3. Experimental

### 3.1. Reagents

CDDO-Me was obtained from the National Cancer Institute, Bethesda, MD, through the Rapid Access to Intervention Development Program. Anti-caspase-3, caspase-8, and caspase-9 antibodies were purchased from BD Pharmingen (San Diego, CA, USA). Antibodies against Akt, p-Akt (ser^473^), mTOR, p-mTOR (Ser^2448^), S6K1, p-S6K1 (thr^421^/ser^424^), 4E-BP1, p-4E-BP1 (thr^37/46^), p-eIF4E (ser^209^), p-Foxo3a (ser^2531^), and cyclin D1 were purchased from Santa Cruz Biotechnology, Inc. (Santa Cruz, CA). CellTiter 96 AQueous One Solution Proliferation Assay (MTS) system was obtained from Promega (Madison, WI, USA).

### 3.2. Cell Lines

Human pancreatic cancer cell lines MiaPaca2, Panc1, Capan2 and BxPC3 were obtained from the American Type Culture Collection (ATCC), Rockville, MD, USA. All cell lines were cultured at 37 °C in a humidified atmosphere consisting of 5% CO_2_ and 95% air and maintained by subculturing cells twice a week.

### 3.3. MTS Assay

Cells (1 × 10^4^) were seeded into each well of a 96-well plate in 100 μL of tissue culture medium. After 24 h incubation to allow cells to adhere, cultures were treated with CDDO-Me for 72 h. Cell viability was then determined by the colorimetric MTS assay using CellTiter 96 AQueous One Solution Proliferation Assay System.

### 3.4. Apoptosis Assay

Apoptosis was assessed by the binding of annexin V-FITC to phosphotidylserine, which is externalized to the outer leaflet of the plasma membrane early during induction of apoptosis. Briefly, untreated cells and cells treated with CDDO-Me for 24 h were resuspended in the binding buffer provided in the annexin V-FITC apoptosis detection kit II (BD Biosciences, San Diego, CA, USA) and reacted with 5 μL of annexin V-FITC reagent and 5 μL of propidium iodide (PI) for 30 min at room temperature in the dark. Stained cells were analyzed by flow cytometry.

### 3.5. Mitochondrial Depolarization Assay

Mitochondrial potential was determined using mitochondrial potential sensor JC-1 (Molecular Probes, Invitrogen, San Diego, CA, USA). 1 × 10^6^ control cells or cells treated with CDDO-Me for 24 h were loaded with JC-1 (10 µg/mL) for 10 minutes at 22 °C and analyzed by flow cytometry. In normal cells, dye is aggregated in mitochondria, fluoresces red, and is detected in the FL2 channel. In cells with altered mitochondrial potential, the dye fails to accumulate in the mitochondria, remains as monomers in the cytoplasm, fluoresces green, and is detected in the FL1 channel.

### 3.6. Western Blotting

Total cellular proteins were isolated by detergent lysis (1% Triton-X 100 (v/v), 10 mM Tris-HCl (pH 7.5), 5 mM EDTA, 150 mM NaCl, 10% glycerol, 2 mM sodium vanadate, 5 μg/mL leupeptin, 1 μg/mL aprotinin, 1 μg/mL pepstatin A, and 10 μg/mL 4-2-aminoethyl-benzenesulfinyl fluoride). Lysates were clarified by centrifugation at 14,000× g for 10 min at 4 °C and protein concentrations were determined by Bradford assay. Protein samples (50 μg) were separated on 10–14% SDS-polyacrilamide gels. Proteins resolved on the gels were transferred to nitrocellulose membranes and probed with protein specific antibodies followed by HRP-conjugated secondary antibody.

### 3.7. DNA Transfection

For silencing of Akt or mTOR, tumor cells were transfected with double stranded siRNA of Akt or mTOR using SignalSilence siRNA kit from Cell Signaling Technology (Beverly, MA, USA). Briefly, 10^6^ cancer cells were plated in 60 mm Petri dish for 24 h and treated with 3 ml of transfection medium containing 20 μg LipofectAMINE and 100 nM siRNA for 24 h. Gene silencing in transfected cells was confirmed by Western blotting.

### 3.8. Statistical Analysis

Data are expressed as means ± S.D.

## 4. Conclusions

Clearly, there is a tremendous need for developing safe and effective therapeutics for pancreatic cancer. CDDO-Me showed a potent growth inhibitory activity for pancreatic cancer cells by inducing apoptosis through both mitochondrial and non-mitochondrial pathways. Pro-survival Akt/mTOR signaling axis appears to be the molecular target for the growth inhibitory and apoptosis-inducing activity of CDDO-Me. These mechanism based pre-clinical studies provide strong support for clinical evaluation of CDDO-Me for pancreatic cancer.
